# Design and implementation of blood donor sample bioarchives to enhance preparedness for emerging and pandemic pathogens in England

**DOI:** 10.2807/1560-7917.ES.2025.30.44.2500163

**Published:** 2025-11-06

**Authors:** Shannah Secret, Peter Simmonds, Janie Olver, Piya Rajendra, Eilish Hart, Abigail A Lamikanra, Hoi Pat Tsang, Niel Garrett, Claire Reynolds, David J Roberts, Susan R Brailsford, Amanda Semper, Heli Harvala

**Affiliations:** 1 Microbiology services, NHS Blood and Transplant, Colindale, United Kingdom; 2 Radcliffe Department of Medicine, University of Oxford, Oxford, United Kingdom; 3 Nuffield Department of Medicine, University of Oxford, Oxford, United Kingdom; 4 Rare and Imported Pathogens Laboratory, UK Health Security Agency, Porton Down, Wiltshire, United Kingdom; 5 National Institute for Health and Social Care Research Health Protection Research Unit in Emerging and Zoonotic Infections, Wiltshire, United Kingdom; 6 Institute of Infection, Veterinary and Ecological Sciences, University of Liverpool, Liverpool, United Kingdom; 7 Diagnostics & Pathogen Characterisation, UK Health Security Agency, Porton Down, Wiltshire, United Kingdom; 8 Clinical Services, NHS Blood and Transplant, Oxford, United Kingdom; 9 Radcliffe Department of Medicine and BRC Haematology Theme, University of Oxford, Oxford, United Kingdom; 10 NHSBT Filton Testing/Blood Supply, Filton, Bristol, United Kingdom; 11 NHS Blood and Transplant/ UK Health Security Agency Epidemiology Unit, NHS Blood and Transplant, London, United Kingdom; 12 Institute of Biomedicine, Faculty of Medicine, University of Turku and Turku University Hospital, Turku, Finland

**Keywords:** West Nile Virus, Tick-borne encephalitis virus, Blood donor, Bioarchive, Pandemic response, Emerging infection, England, blood-borne infections, vector-borne infections, bacterial infections, viral infections, tick-borne encephalitis - TBE, West Nile virus, outbreaks, laboratory surveillance, sentinel surveillance

## Abstract

New and emerging infections with blood-borne pathogens pose an ongoing threat to the safety of blood transfusions and transplants. Bioarchives of stored blood donor samples represent a valuable pathogen screening resource for both ensuring safety of blood transfusions and for wider public health infectious disease surveillance. Large scale testing of donors enables early detection of pathogen spread and extent of population exposure. We have implemented two complementary systems for the bioarchiving of blood donor samples in England for these purposes. The CODONET bioarchive collects samples from geographically targeted regions of potential pathogen emergence. Consenting donors provide detailed information to allow for risk assessment and, importantly, to distinguish imported from autochthonously acquired infection. Separately, the blood donor surveillance archive (BDSA) stores 100 or 200 pools of 24 randomly selected, fully anonymised donation samples from donors in England every 2 weeks, allowing large-scale continuous sampling. This enables rapid evaluation of the presence of blood-borne pathogens in donor populations and a large-scale epidemiological tool to detect pathogen emergence in real-time. Combined, these bioarchives allow for proactive assessment of donation transmission risk, and as targeted population-wide archives, contribute to public health surveillance of emerging pathogens and pandemic spread.

## Background

National Health Service Blood and Transplant (NHSBT) is a large blood service responsible for the collection, screening and provision of ca 2 million red cell and other blood component units per year to hospitals for transfusion in England. Transmission of HIV, hepatitis B (HBV), hepatitis C (HCV) and hepatitis E (HEV) virus and of bacteria in platelets have been virtually eliminated since the introduction of stringent screening to ensure blood safety. However, ongoing horizon scanning and risk assessments, not only in England but also in the European Union (EU) and United States (US), are pivotal for identifying and mitigating any potential future microbiological risk threatening blood safety [1].

Outbreaks and pandemics caused by infectious pathogens are not new; in the past 100 years, human populations have been subject to recurrent and often devastating plagues and virus outbreaks caused by smallpox, measles and coronaviruses, as well as retroviruses such as HIV-1 and Ebola virus in West Africa, and many pandemic influenza A viruses (IAV) (reviewed in reference [2]). In addition, the global outbreaks of dengue virus (DENV), especially in South East Asia and the Americas with local introduction in Europe, and the very recent Oropouche virus outbreaks in South America exemplify the change in circulation of arboviruses. This change follows the spread of mosquito or tick vectors in response to multiple factors including climate change and human activities such as international travel and trade [3-5].

In Europe, the recent emergence of infections with West Nile virus (WNV) in Germany and France has been associated with the presence of *Culex* mosquito vectors [6]. West Nile virus became permanently established in certain parts of Germany from 2018 [6] and blood donor screening in endemic areas in Germany was implemented in 2020 [7]. Of concern for United Kingdom (UK) blood safety was the detection of WNV in two pools of native *Aedes vexans* mosquitoes collected in 2023 near Nottingham in mid-England [8]. In addition, there are reports that *Culex modestus* has recently become established in the south-east of England and may act as a bridge vector for the introduction of WNV from continental Europe [9]. There is a further potential threat from the related Usutu virus (USUV) whose vector, *Culex pipiens* is established in the UK and USUV-infected birds and mosquitoes have been reported in the London area [10].

The recent local expansion of DENV infections in France, Italy and Spain [11] is potentially worrisome should the *Aedes albopictus* vector range expand further northwards and over winter in the UK. There has been a parallel expansion of tick-borne diseases, including tick-borne encephalitis (TBE) and Crimean-Congo haemorrhagic fever, into new areas of Europe. Since 2012, reported cases of TBE have increased in incidence and geographical range across the European continent even though suspected or confirmed cases of transfusion-transmitted infections remain rare exceptions [12]. The first probable case of tick-borne encephalitis virus (TBEV) acquired in England was in 2019 [13] followed by two laboratory-confirmed cases of TBEV with likely UK acquisition in 2022 [14]. The first case presented with encephalitis (TBE), but no clinical data were available for the next two TBEV cases. Surveillance of sentinel animals across England and targeted surveillance of *Ixodes ricinus* ticks has identified enzootic foci of TBEV in several areas [15].

Measures taken by many countries towards ‘pandemic preparedness’ demonstrate a commitment towards establishing a more effective and pre-specified pathway to respond to future public health crises. Surveillance and protection of the blood supply represents a major component of such planning. Here we describe the development and set-up of two sample archiving programmes that will provide a novel and robust infrastructure with which to proactively assess threats to blood safety from emerging pathogens.

## Creation of a Donation Bioarchive to Study and Assess the Spread of Newly Emerging Pathogens (CODONET)

The first archive programme, Creation of a Donation Bioarchive to Study and Assess the Spread of Newly Emerging Pathogens (CODONET), enables geographically targeted collection and archiving of well-characterised donor samples for surveillance purposes. In England, blood donors are enrolled through email invitation to participate in the study and complete a consent form before responding to an electronic survey created and operated by the UK Health Security Agency (UKHSA). The survey collects detailed demographic information and medical and vaccination histories from the donors to facilitate interpretation of results and determine risk factors for infection. Detailed information on contact with animals and vectors and outdoor occupational and recreational activities is also collected for investigation of zoonotic infections (Table 1; the full form is provided in Supplement S1). Once a donor has consented, ca 2 ml of residual plasma sample from their next routine blood donation is retained after routine testing and added in multiple aliquots to the CODONET bioarchive.

**Table 1:** Abbreviated summary of donor information collected in the Creation of a Donation Bioarchive to Study and Assess the Spread of Newly Emerging Pathogens (CODONET) questionnaire^a^, England

Donor characteristics	Information parameters
Age	Age (in years)
Biological sex, gender	Male / female and gender identify
Ethnic group	Asian or Asian British
	Black, Black British, Caribbean or African
	Mixed or multiple ethnic groups
	Other
	White
Residency	Current residential postcode
	Duration of residence
	Country of birth (if not UK)
	Other countries of residence
Travel	Travel outside the UK in the past 5 years: Continents and countries visited, frequency, urban or rural areas
Vaccinations	Vaccinations for unusual or travel-related diseases Vaccine type: Tick-borne encephalitis, yellow fever, Japanese encephalitis, dengue fever, Q fever, rabies, hepatitis A, hepatitis B, Lyme disease (vaccine discontinued), smallpox, monkeypox
	Timing of most recent vaccination
Medical history	Previous deferral from blood donation
	Previous zoonotic diseases and recency of diagnosis
Exposure to ticks	History of tick bites in past 5 years
	Location(s) of tick bites, UK, abroad
	How frequently bitten and frequency of exposure to areas with high numbers of ticks
Exposure to mosquitoes	History of mosquito bites in past 5 years
	Location(s) of mosquito bites, UK, abroad
	How frequently bitten and frequency of exposure to areas with high numbers of mosquitoes
Exposure to animals	History of animal exposure in past 10 years
	Reason and frequency of contact with wild, farm or pet species: Pigs, cattle, sheep, goats, deer, poultry, gamebirds, wild birds, pet birds, camels, llamas, alpacas, bats, dogs, cats, rabbits, guinea pigs, mice, rats, hamsters, gerbils, reptiles, horses, non-human primates, insects, fish
	History of animal bites
Food products	List of dietary exclusions
	Consumption of unpasteurised milk
Outdoor exposure	Outdoor employment in past 10 years, including: Wildlife ranger, deer management, forestry worker, forestry ranger, park ranger, countryside management, conservation, gamekeeper, gamebird management, professional beater, farm worker, farm manager, small holder, gardener, outdoor pursuits instructor, environmental scientist, field ecologist, veterinarian, water worker, fisherman, other
	Duration of outdoor occupation(s)
	Outdoor hobbies or leisure activities in past 10 years, including: Urban walking or running, rural walking or running, urban dog walking, rural dog walking, orienteering, climbing, bouldering, cycling (road or off-road), horse riding, golf, field sport (cricket, football, rugby), gardening, camping (including attending festivals),forest or conservation volunteer, recreational shooting or hunting, recreational deer stalking or beating, freshwater sports (swimming, sailing, rowing, caving, fishing), sea sports, other
	Duration of outdoor activities

UK: United Kingdom.

^a^ Complete form provided in Supplement S1.

In addition to quantifying past or current infection frequencies in blood donors, detailed data collected on donors through CODONET will help determine if cases are locally acquired or acquired through international travel. Information is also collected on specific risk factors for infections, such as age, biological sex, gender and pursuit of outdoor activities that may increase exposure to mosquitoes or ticks, as well as information on vaccination history. These collected data will be essential for interpreting the serosurveillance data for viruses such as TBEV and for assessing and identifying risk factors. As appropriate, the CODONET framework also allows for donors to be recalled, resampled and reinterviewed to verify information provided when evaluating results that are potentially important to the blood supply or public health. Follow-up of recipients will be organised via NHSBT if results with potential clinical or public health importance are identified.

One initial application of the CODONET archive will be to determine whether WNV, and USUV infections are becoming established in areas of England and present a risk for autochthonous infections in humans. To date, a total of 42,000 regular donors in NHSBT donor centres in the appropriate postcodes have been identified and will be invited to take part in the CODONET programme to identify serological evidence for exposure to WNV (Figure; a full list of postcodes is appended in Supplementary Table S1A; Supplement S1). Those with confirmed serological evidence for past WNV infections analysed by ELISA and neutralising antibody testing, will be related to demographic, vaccination and vector or animal exposure information from donors to assess the occurrence of potential locally acquired infections.

**Figure:**
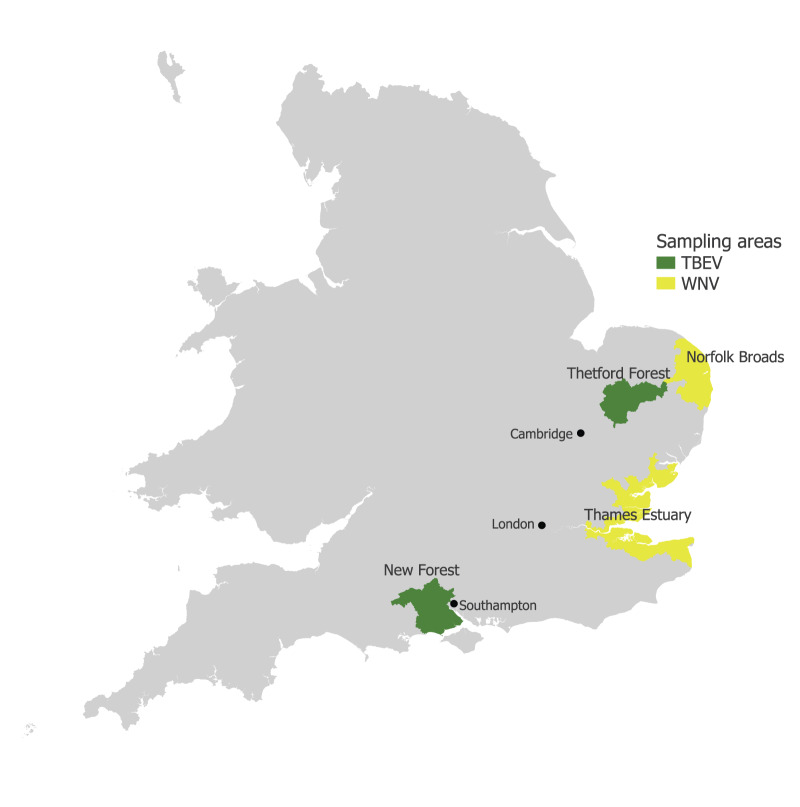
Regions sampled for West Nile virus and tick-borne encephalitis virus serosurveillance, England, 2024–2025

TBEV: tick-borne encephalitis virus; WNV: West Nile virus.

A second planned application of the CODONET archive is to determine when and where new tick-borne infections are emerging in England, such as TBEV. To prepare for this, we have initiated targeted donor sample collections in these two areas (Figure; green shaded areas; Supplementary Table S1B, Supplement S1). Samples will be analysed serologically to assess the occurrence of potential locally acquired TBEV infections, including those which may have been asymptomatic and have therefore gone undetected. IgG seroprevalence estimates in blood donors from these ‘tick-risk’ areas will be compared with blood donor populations elsewhere in southern England where tick exposure risk is lower (such as the regions identified as being at risk for WNV and USUV; Figure).

## Blood Donor Surveillance Archive

Bioarchiving of surplus plasma volumes of mini-pools derived from 24 blood and platelet donations used for routine donation screening by nucleic acid testing (NAT) for HIV, HCV, HBV and HEV has been initiated. Between 100 and 200 mini-pools (representing 2,400 and 4,800 donors, respectively) are collected every 2 weeks and are anonymised and unlinked before shipment to the NHSBT bioarchive. Pools and their component donations cannot be linked to donors but provide a resource to rapidly determine population-level viraemia and seroprevalences to emerging infections or novel pathogens in a pandemic situation, with the necessary restriction on analytical sensitivity for samples with low pathogen loads. Between 10 May 2023 and 28 February 2025, a total of 4,097 pools corresponding to 98,328 donor samples had been archived. Complementary roles and differences between the Blood Donor Surveillance Archive (BDSA) and CODONET bioarchives are presented in Table 2.

**Table 2:** Complementary roles and differences between the Creation of a Donation Bioarchive to Study and Assess the Spread of Newly Emerging Pathogens (CODONET) and the Blood Donor Surveillance Archive bioarchives, England

Archive type	CODONET (Creation of a Donation Bioarchive to Study and Assess the Spread of Newly Emerging Pathogens)	BDSA (Blood Donor Surveillance Archive)
Purpose	Targeted testing of donors. With ability to focus recruitment or testing according to risk factors, travel history and vector exposure	Rapid, large-scale untargeted molecular and serosurveillance population studies in response to emerging or pandemic pathogens
Strengths	Additional donor data allow for identification of autochthonous cases, risk factors and epidemiological interpretation	Provides rapid evaluation of the spread or population exposure to emerging infections in a large sample base
Sample type	Surplus plasma from donor’s next blood donation	Surplus plasma from mini-pools of 24 donors
Collection frequency	Continuous recruitment of donors. Sample collected when consented donor next donates	100 (winter) or 200 mini-pools (summer) from 2,400 to 4,800 randomly selected donors every 2 weeks
Data collected	Donor completes questionnaire providing demographic data, vaccine, travel, occupational and exposure history which is linked to the sample	None, beyond the standard collection every 2 weeks
Targeted recruitment	Yes. Email recruitment to active blood donors. Geographical or demographic targeting possible e.g. donors living in areas of known vector presence	No. Random national sampling
Sample linking and anonymisation	Pseudoanonymised. Can link back to donor for recall or further testing or questioning	Full anonymisation. Not possible to link back to donor
Consent	Explicit consent to participate provided electronically	Consent is covered by general NHSBT donor consent
Ethics	Ethical approval from the NW England Regional Ethics committee (reference 24/NW/0127)	Approved internally by NHSBT (BS-CARE, NCI)
Scalability	Staff resources limit collections to ca 5,000 samples per year	Readily scaled-up to all donors in England as required
Collection started	December 2024	May 2023

BS-CARE: Blood Supply Clinical Audit, Risk and Effectiveness Committee of NHSBT; NCI: Non-Clinical Issue; NHS: National Health Service; NHSBT: National Health Service Blood and Transplant; NW: North West.

## Bioarchives for hemovigilance and as a public health resource

We plan prospective long-term maintenance of the BDSA and CODONET bioarchives to ensure that archived sample resources are in place should another pandemic or major pathogen emergence occur in the future. For example, large serosurveillance and epidemiological studies on severe acute respiratory syndrome coronavirus 2 (SARS-CoV-2) were conducted during the COVID-19 pandemic using residual blood donation samples [16]. The bioarchives also provide a resource for evidence-based assessments of microbiology transmission risk arising from horizon-scanning by the Standing Advisory Committee for Transfusion Transmitted Infections (SACTTI) and other hemovigilance activities in the UK and elsewhere. In addition, these archives are not limited to the study of only infectious agents that are a threat to blood safety but can also be used for wider public health surveillance. Specifically, we demonstrated the lack of monkeypox virus (MPXV) circulation among the blood donor population using the mini-pools for MPXV DNA testing following the emergence of MPXV in the UK and in other western countries in April–May 2022 predominantly among certain groups of gay, bisexual and other men who have sex with men (GBMSM) [17].

Among other potential emerging pathogen threats, the archives can potentially be used to investigate human circulation of high pathogenicity strains of H5N1 IAV, following the description of more than 60 human cases in late 2024 and one death [18]. While experience from the swine-origin H1N1 IAV pandemic in 2009 suggests that IAV is not a specific threat to blood safety, with asymptomatic donors showing no evidence of viraemia [19,20], infections with the more pathogenic H5N1 in the prodromal stage may show greater systemic spread and viraemia frequencies. The BDSA and CODONET archives are of further potential value as a resource to monitor population exposure and timing through the application of H5N1-specific serology assays.

## Establishment of the bioarchives

To maximise their operational simplicity, both bioarchives were designed at the outset to avoid additional donor sampling by using leftover samples and to avoid imposing additional workloads at donor centres through the use of online consent and survey completion by donors before donation (CODONET). The principal operational complexity of the CODONET bioarchive was the completion of internal risk-assessments, documentation and contracts with collaborators at UKHSA. In addition to data governance and information storage, consent and survey information provided by the donor had to be linked to their donation sample while preserving anonymity through recoding. Sampling can be targeted based on the geographical location of the identified risk, and similarly, the questionnaire can be modified to cover the suspected risk.

## Conclusion

We believe that the establishment of CODONET and BDSA bioarchives and a capacity for large-scale and rapid screening for novel and emerging pathogens represent a concrete and decisive step towards preparedness for the next emerging or pandemic pathogen. For most emerging infections, blood donors are reasonably representative of the general population and are of considerable value as a sentinel group used to monitor population exposure to emerging and future potential pandemic pathogens. However, we acknowledge the blood donor population is not fully representative of the population; limitations include the age range restriction of donors (17 to around 70 years), under-representation of some ethnic minorities and of donors at risk for sexually-transmitted infections including MPXV, where potential donors reporting high-risk sexual activity are excluded [21]. The dilution of samples in mini-pools of 24 limits the sensitivity of testing archive samples with low viral loads or antibody titres. Nevertheless, the use of the two bioarchives to provide the earliest possible data on the spread of novel and emerging pathogens and the safety of the blood supply will inform the national public health response on aspects that were incomplete or missing in previous outbreaks and pandemics. Establishing these bioarchives also addresses potential shortcomings in the UK blood services in terms of capacity to respond rapidly and proactively to emerging pathogens, as recently highlighted by the UK Infected Blood Inquiry [22].

## Data Availability

All data presented in the manuscript or supplementary material are available from the corresponding author upon reasonable request.

## Supplementary Data

554000 Supplementary Material

## Supplementary Data

1609000 Supplementary Table
