# Supplementary material for: Design and implementation of blood donor sample bioarchives to enhance preparedness for emerging and pandemic pathogens in England
Source: Euro Surveill. 2025 Nov 6;30(44):2500163. doi: 10.2807/1560-7917.ES.2025.30.44.2500163 (PMC12595290; doi:10.2807/1560-7917.ES.2025.30.44.2500163)
Supplement: Supplementary Material [file 25-00163_HARVALA_SuplementS1.pdf]

This supplementary material is hosted by *Eurosurveillance* as supporting information alongside the article 'Design and implementation of blood donor sample bioarchives to enhance preparedness for emerging and pandemic pathogens in England', on behalf of the authors, who remain responsible for the accuracy and appropriateness of the content. The same standards for ethics, copyright, attributions and permissions as for the article apply. Supplements are not edited by *Eurosurveillance* and the journal is not responsible for the maintenance of any links or email addresses provided therein.

Emerging Infections in England

CODONET Study Questionnaire (Version 3 - 06/11/24)

You have been asked to complete this questionnaire as part of the CODONET study.
Your responses will help us to investigate current and emerging infections in England and
potential risk factors.

*Please complete this questionnaire **before** attending your donation session. If you have donated before, please try to attend a donation session in your normal area.*

The questionnaire asks about:
- your age, gender & ethnicity
- where you live
- overseas travel in the last 5 years
- 'travel' vaccinations you've had in the past (e.g. yellow fever, hepatitis A)
- any tropical or unusual diseases you've caught in the past
- any known exposures to ticks or mosquitoes
- your exposure to animals
- your occupation if you work outdoors
- your outdoor hobbies & leisure activities

You may find it helpful to check some of this information before you start to answer the questionnaire.

NHS
Blood and Transplant

UK Health
Security
Agency

How to fill in the questionnaire

Where possible, use a desktop, laptop or tablet
The questionnaire will work on a Smartphone but you may have to scroll down your screen to find the Back / Next buttons

Use the 'Next' and 'Back' buttons at the bottom of each page not the back button on your browser.

If you are using a mobile and you receive the message 'page not working', please refresh your page to reload the questionnaire.

If you need to save a partially completed questionnaire, use the **'Save'** button. You will be sent an email with a link so that you can return and complete the questionnaire later.

If you have any problems with this questionnaire, please contact CODONET@ukhsa.gov.uk

Personal details

What is your age (in years)?

123

What is your biological sex, as assigned at birth?
Please select ONE option. A voluntary question about gender identity will follow

- ◯ Female
- ◯ Male

Is the gender you identify with the same as your sex registered at birth? ***This question is voluntary***

- ◯ Yes
- ◯ No

If No, please state the gender you identify with

Choose one option that best describes your ethnic group or background. You can give more detail in the next question

- ○ White
- ○ Mixed or multiple ethnic groups
- ○ Asian or Asian British
- ○ Black, Black British, Caribbean or African
- ○ Prefer not to answer
- ○ Other

You selected 'White'. Now please choose the option that best describes you

- English, Welsh, Scottish, Northern Irish or British
- Irish
- Gypsy or Irish Traveller
- Roma
- Prefer not to say
- Any other white background, please specify

You selected 'Mixed or multiple ethnic groups'. Now please choose the option that best describes you

- White and Black Caribbean
- White and Black African
- White and Asian
- Prefer not to say
- Any other Mixed or multiple ethnic background, please specify

You selected 'Asian or Asian British'. Now please choose the option that best describes you

- Indian
- Pakistani
- Bangladeshi
- Chinese
- Prefer not to say
- Any other Asian background, please specify

You selected 'Black, African, Caribbean or Black British'. Now please choose the option that best describes you

- African
- Caribbean
- Prefer not to say
- Any other Black, African or Caribbean background, please specify

You selected 'Other ethnic group'. Now please choose the option that best describes you

- Prefer not to say
- Any other ethnic group, please specify

Residency

The CODONET study is looking in the blood for evidence of exposure to new and current infections.
The following questions will help us understand if exposure is likely to have occurred in the UK or abroad.

What is the full postcode of your usual residence in the UK?

____

How many years have you lived at this postcode (approximately)?

- ○ Less than 1 year
- ○ 1 to 5 years
- ○ 5 to 10 years
- ○ More than 10 years

How many years have you lived uninterrupted in the UK? Please give your best guess if you can't remember exactly. Do not count short periods (one month or less) away from the UK.

123 __________

Were you born in the UK?

- ○ Yes
- ○ Prefer not to say
- ○ No

Please specify your country of birth

____

Were you resident in your country of birth for **more than 6 months**?

- ○ Yes
- ○ No
- ○ Not sure / can't remember

Other than your birth country or the UK, have you lived in any other country/countries for **more than 6 months**?

- ○ No
- ○ Not sure / can't remember
- ○ Yes, please specify country or countries

Overseas travel

In the **last 5 years,** have you travelled outside the UK e.g. for business, leisure or visiting friends and/or family?

- ◯ Yes — Go to Q18
- ◯ No — Go to N11

In the **last 5 years,** how many times have you travelled outside the UK? **Do not include transit or stopovers where you did not leave the airport or port.**

- ◯ 1-10 times
- ◯ 11-20 times
- ◯ 21-30 times
- ◯ More than 30 times

Please tick the areas you travelled to in the last 5 years. *Select all that apply*

- ☐ Africa
- ☐ Asia (including the Middle East, Turkey, Russia and China)
- ☐ North America
- ☐ South America (including Central America and the Caribbean)
- ☐ Europe
- ☐ Oceania (including Australia, New Zealand and the Pacific Islands)
- ☐ Antarctica

Please list the countries in Africa you have spent time in over the **last 5 years**

What areas did you stay in during your visits to Africa? *Select all that apply*

- ☐ Urban (i.e. cities or towns)
- ☐ Rural (i.e. villages or countryside)

Please list the countries in Asia you have spent time in over the **last 5 years**

What areas did you stay in during your visits to Asia? *Select all that apply*

- ☐ Urban (i.e. cities or towns)
- ☐ Rural (i.e. villages or countryside)

Please list the countries in North America you have spent time in over the **last 5 years**

What areas did you stay in during your visits to North America? *Select all that apply*

- ☐ Urban (i.e. cities or towns)
- ☐ Rural (i.e. villages or countryside)

Please list the countries in South America you have spent time in over the **last 5 years**

What areas did you stay in during your visits to South America? *Select all that apply*

- ☐ Urban (i.e. cities or towns)
- ☐ Rural (i.e. villages or countryside)

Please list the countries in Europe you have spent time in over the **last 5 years**

What areas did you stay in during your visits to Europe? *Select all that apply*

- ☐ Urban (i.e. cities or towns)
- ☐ Rural (i.e. villages or countryside)

Please list the countries in Oceania you have spent time in over the **last 5 years**

What areas did you stay in during your visits to Oceania? *Select all that apply*

- ☐ Urban (i.e. cities or towns)
- ☐ Rural (i.e. villages or countryside)

Vaccinations

This question is about vaccinations you may have been given **for unusual or travel-related diseases.**

You may have been vaccinated:
- as part of a national vaccination programme if you lived outside the UK
- before travelling abroad
- because of your job e.g. as a member of the armed forces or as a healthcare worker

To your knowledge, have you ever had any of the following vaccinations or booster vaccinations? *Select all that apply*

- [ ] Tick-borne encephalitis (TBE)
- [ ] Yellow fever
- [ ] Japanese encephalitis (JE)
- [ ] Dengue fever
- [ ] Q fever (aka Coxiella)
- [ ] Rabies
- [ ] Hepatitis A
- [ ] Hepatitis B
- [ ] Lyme disease (vaccine discontinued)
- [ ] Smallpox
- [ ] Monkeypox (mpox)
- [ ] None of these vaccinations

Please check your answer. You cannot select 'None of these vaccinations' with another option

When did you last have a vaccination for tick-borne encephalitis?

- Within the last month
- Within the last 6 months
- Within the last year
- Within the last 5 years
- Within the last 10 years
- More than 10 years ago
- Unsure

When did you last have a vaccination for yellow fever?

- Within the last month
- Within the last 6 months
- Within the last year
- Within the last 5 years
- Within the last 10 years
- More than 10 years ago
- Unsure

When did you last have a vaccination for Japanese encephalitis?

- Within the last month
- Within the last 6 months
- Within the last year
- Within the last 5 years
- Within the last 10 years
- More than 10 years ago
- Unsure

When did you last have a vaccination for Dengue fever?

- Within the last month
- Within the last 6 months
- Within the last year
- Within the last 5 years
- Within the last 10 years
- More than 10 years ago
- Unsure

When did you last have a vaccination for Q fever (also known as Coxiella)?

- Within the last month
- Within the last 6 months
- Within the last year
- Within the last 5 years
- Within the last 10 years
- More than 10 years ago
- Unsure

When did you last have a vaccination for rabies?

- Within the last month
- Within the last 6 months
- Within the last year
- Within the last 5 years
- Within the last 10 years
- More than 10 years ago
- Unsure

When did you last have a vaccination for hepatitis A?

- Within the last month
- Within the last 6 months
- Within the last year
- Within the last 5 years
- Within the last 10 years
- More than 10 years ago
- Unsure

When did you last have a vaccination for hepatitis B?

- Within the last month
- Within the last 6 months
- Within the last year
- Within the last 5 years
- Within the last 10 years
- More than 10 years ago
- Unsure

When did you last have a vaccination for Lyme disease?

- Within the last month
- Within the last 6 months
- Within the last year
- Within the last 5 years
- Within the last 10 years
- More than 10 years ago
- Unsure

When did you last have a vaccination for smallpox?

- Within the last month
- Within the last 6 months
- Within the last year
- Within the last 5 years
- Within the last 10 years
- More than 10 years ago
- Unsure

When did you last have a vaccination for monkeypox (mpox)?

- Within the last month
- Within the last 6 months
- Within the last year
- Within the last 5 years
- Within the last 10 years
- More than 10 years ago
- Unsure

Medical history

Have you ever been told not to give blood due to infections in the past?

- ◯ No
- ◯ Not sure / prefer not to say
- ◯ Yes

Please state what infection you had that meant you were asked not to give blood

To your knowledge, have your **ever** been diagnosed by a medical professional with any of the following diseases? *Select all that apply*

- ☐ Lyme disease / Lyme borreliosis / neuroborreliosis
- ☐ Tick-borne encephalitis (TBE)
- ☐ Dengue fever
- ☐ Zika / Zika fever
- ☐ Yellow fever
- ☐ Japanese encephalitis (JE)
- ☐ West Nile fever
- ☐ Chikungunya
- ☐ Hepatitis A
- ☐ Hepatitis B
- ☐ Leptospirosis / Weil's disease
- ☐ Q fever (Coxiella)
- ☐ Usutu
- ☐ Anaplasmosis (HGA)
- ☐ Babesiosis
- ☐ Ehrlichia / Ehrlichiosis
- ☐ None of these

Please check your answer. You cannot select 'None of these' with another option

You stated you have previously been diagnosed with Lyme disease. How recently did you receive this diagnosis?

- Within the last month
- Within the last 6 months
- Within the last year
- Within the last 5 years
- Within the last 10 years
- More than 10 years ago

You stated you have previously been diagnosed with Tick-borne encephalitis (TBE). How recently did you receive this diagnosis?

- Within the last month
- Within the last 6 months
- Within the last year
- Within the last 5 years
- Within the last 10 years
- More than 10 years ago

You stated you have previously been diagnosed with Dengue fever. How recently did you receive this diagnosis?

- Within the last month
- Within the last 6 months
- Within the last year
- Within the last 5 years
- Within the last 10 years
- More than 10 years ago

You stated you have previously been diagnosed with Zika / Zika fever. How recently did you receive this diagnosis?

- Within the last month
- Within the last 6 months
- Within the last year
- Within the last 5 years
- Within the last 10 years
- More than 10 years ago

You stated you have previously been diagnosed with Yellow fever. How recently did you receive this diagnosis?

- Within the last month
- Within the last 6 months
- Within the last year
- Within the last 5 years
- Within the last 10 years
- More than 10 years ago

You stated you have previously been diagnosed with Japanese encephalitis. How recently did you receive this diagnosis?

- Within the last month
- Within the last 6 months
- Within the last year
- Within the last 5 years
- Within the last 10 years
- More than 10 years ago

You stated you have previously been diagnosed with West Nile fever. How recently did you receive this diagnosis?

- Within the last month
- Within the last 6 months
- Within the last year
- Within the last 5 years
- Within the last 10 years
- More than 10 years ago

You stated you have previously been diagnosed with Chikungunya. How recently did you receive this diagnosis?

- Within the last month
- Within the last 6 months
- Within the last year
- Within the last 5 years
- Within the last 10 years
- More than 10 years ago

You stated you have previously been diagnosed with Hepatitis A. How recently did you receive this diagnosis?

- Within the last month
- Within the last 6 months
- Within the last year
- Within the last 5 years
- Within the last 10 years
- More than 10 years ago

You stated you have previously been diagnosed with Hepatitis B. How recently did you receive this diagnosis?

- Within the last month
- Within the last 6 months
- Within the last year
- Within the last 5 years
- Within the last 10 years
- More than 10 years ago

You stated you have previously been diagnosed with Leptospirosis / Weil's disease. How recently did you receive this diagnosis?

- Within the last month
- Within the last 6 months
- Within the last year
- Within the last 5 years
- Within the last 10 years
- More than 10 years ago

You stated you have previously been diagnosed with Q fever (Coxiella). How recently did you receive this diagnosis?

- Within the last month
- Within the last 6 months
- Within the last year
- Within the last 5 years
- Within the last 10 years
- More than 10 years ago

You stated you have previously been diagnosed with Usutu. How recently did you receive this diagnosis?

- Within the last month
- Within the last 6 months
- Within the last year
- Within the last 5 years
- Within the last 10 years
- More than 10 years ago

You stated you have previously been diagnosed with Anaplasmosis (HGA). How recently did you receive this diagnosis?

- Within the last month
- Within the last 6 months
- Within the last year
- Within the last 5 years
- Within the last 10 years
- More than 10 years ago

You stated you have previously been diagnosed with Babesiosis. How recently did you receive this diagnosis?

- Within the last month
- Within the last 6 months
- Within the last year
- Within the last 5 years
- Within the last 10 years
- More than 10 years ago

You stated you have previously been diagnosed with Ehrlichia / Ehrlichiosis. How recently did you receive this diagnosis?

- Within the last month
- Within the last 6 months
- Within the last year
- Within the last 5 years
- Within the last 10 years
- More than 10 years ago

Exposure to ticks

Ticks can transmit a variety of diseases to humans when they bite and take a blood meal e.g. Lyme disease, tick-borne encephalitis

The following questions ask about your **exposure to ticks**. This may have been either in the UK or abroad

In the **last 5 years**, are you aware of spending time in areas with a high number of ticks either in the UK or abroad? *Select all that apply*

- ☐ Yes, in the UK
- ☐ Yes, abroad
- ☐ No
- ☐ Not sure

Please check your answer. You cannot select 'No' with another option

Please check your answer. You cannot select 'Not sure' with another option

In which UK nations were you in an area with a high number of ticks? *Select all that apply*

- ☐ Scotland
- ☐ England
- ☐ Northern Ireland
- ☐ Wales
- ☐ Not sure
- ☐ Other, please specify

____

In the last year, how often on average, have you been in areas with a high number of ticks in the UK?

- ○ Every day
- ○ At least once a week
- ○ At least once a month
- ○ At least once every 6 months
- ○ Only once in the year
- ○ No contact in the last year

You answered 'Yes, abroad'. Please list the country or countries where you were in an area with a high number of ticks.

In the last year, how often on average, have you been in areas with a high number of ticks abroad?

- Every day
- At least once a week
- At least once a month
- At least once every 6 months
- Only once in the year
- No contact in the last year

In the **last 5 years**, are you aware of being ***bitten* by ticks** in the UK or abroad? *Select all that apply*

- ☐ Yes, in the UK
- ☐ Yes, abroad
- ☐ No
- ☐ Not sure

Please check your answer. You cannot select 'No' with another option

Please check your answer. You cannot select 'Not sure' with another option

In which UK nations were you bitten by ticks? *Select all that apply*

- ☐ Scotland
- ☐ England
- ☐ Northern Ireland
- ☐ Wales
- ☐ Not sure
- ☐ Other, please specify

____

In the **last 5 years**, approximately how many times **in total** have you been bitten by a tick **in the UK**?

- ○ 1 to 10 times
- ○ 11 to 50 times
- ○ 51 to 100 times
- ○ More than 100 times

Roughly when was your most recent tick bite **in the UK?**

- ○ Within the last week
- ○ Within the last month
- ○ Within the last 6 months
- ○ Within the last year
- ○ More than 1 year ago

You stated you've been bitten **in the last 5 years** by ticks when **abroad**. Please list the country or countries where you were bitten**.**

In the last 5 years, approximately how many times **in total** have you been bitten by a tick **when abroad**?

- 1 to 10 times
- 11 to 50 times
- 51 to 100 times
- More than 100 times

Roughly when was your most recent tick bite **abroad**?

- Within the last week
- Within the last month
- Within the last 6 months
- Within the last year
- More than 1 year ago

Exposure to mosquitoes

Mosquitoes can transmit a variety of diseases to humans when they bite and take a blood meal e.g. dengue fever, West Nile fever.

The following questions ask about your **exposure to mosquitoes**. This may either have been in the UK or abroad.

In the **last 5 years**, are you aware of spending time in areas with a high number of mosquitoes either in the UK or abroad? *Select all that apply*

- ☐ Yes, in the UK
- ☐ Yes, abroad
- ☐ No
- ☐ Not sure

Please check your answer. You cannot select 'No' with another option

Please check your answer. You cannot select 'Not sure' with another option

In which UK nations were you in an area with a high number of mosquitoes? *Select all that apply*

- ☐ Scotland
- ☐ England
- ☐ Northern Ireland
- ☐ Wales
- ☐ Not sure
- ☐ Other, please specify

____

In the last year, how often on average, have you been in areas with a high number of mosquitoes in the UK?

- ◯ Every day
- ◯ At least once a week
- ◯ At least once a month
- ◯ At least once every 6 months
- ◯ Only once in the year
- ◯ No contact in the last year

You answered 'Yes, abroad'. Please list the country or countries where you were in an area with a high number of mosquitoes.

In the last year, how often on average, have you been in areas with a high number of mosquitoes abroad?

- Every day
- At least once a week
- At least once a month
- At least once every 6 months
- Only once in the year
- No contact in the last year

In the **last 5 years**, are you aware of being ***bitten* by mosquitoes** in the UK or abroad? *Select all that apply*

- ☐ Yes, in the UK
- ☐ Yes, abroad
- ☐ No
- ☐ Not sure

Please check your answer. You cannot select 'No' with another option

Please check your answer. You cannot select 'Not sure' with another option

In which UK nations were you bitten by mosquitoes? *Select all that apply*

- ☐ Scotland
- ☐ England
- ☐ Northern Ireland
- ☐ Wales
- ☐ Not sure
- ☐ Other, please specify

In the **last 5 years**, approximately how many times **in total** have you been bitten by a mosquito **in the UK**?

- ○ 1 to 10 times
- ○ 11 to 50 times
- ○ 51 to 100 times
- ○ More than 100 times

Roughly when was your most recent mosquito bite **in the UK**?

- ○ Within the last week
- ○ Within the last month
- ○ Within the last 6 months
- ○ Within the last year
- ○ More than 1 year ago

You stated you've been bitten **in the last 5 years** by mosquitoes when **abroad**. Please list the country or countries where you were bitten**.**

In the last 5 years, approximately how many times **in total** have you been bitten by a mosquito **when abroad**?

- 1 to 10 times
- 11 to 50 times
- 51 to 100 times
- More than 100 times

Roughly when was your most recent mosquito bite **abroad**?

- Within the last week
- Within the last month
- Within the last 6 months
- Within the last year
- More than 1 year ago

Exposure to animals

The following questions ask if you have contact with animals (alive or dead), their waste products or their bedding. This includes regular contact throughout the year or more intermittent contact that has carried on for several years.

In the **last 10 years**, have you had regular or intermittent contact with animals (alive or dead), their waste products or their bedding ?

○ Yes
○ No

In the **last 10 years**, have you had regular or intermittent contact with any of the following animals (alive or dead), their waste products or their bedding? *Select all that apply*

- [ ] Cats
- [ ] Dogs
- [ ] Rabbits
- [ ] Guinea pigs
- [ ] Rodents (e.g. mice, rats, hamsters, gerbils)
- [ ] Horses
- [ ] Pet birds
- [ ] Reptiles
- [ ] Cattle
- [ ] Pigs
- [ ] Sheep or goats
- [ ] Poultry or reared gamebirds
- [ ] Deer
- [ ] Wild birds
- [ ] Insects
- [ ] Fish
- [ ] Bats
- [ ] Camels, llamas or alpacas
- [ ] Non-human primates (monkeys or apes)
- [ ] Other

Please specify other animals, <u>using a new box for each animal</u> (a total of 10 boxes are available)

[ ]

You indicated you have had regular or seasonal contact with cats in the **last 10 years**. What was the reason for this? *Select all that apply*

- ☐ Work
- ☐ Pet
- ☐ Hobby (e.g. smallholding)
- ☐ Other, please specify

In the last year, how often on average, have you been in contact with cats?

- ○ Every day
- ○ At least once a week
- ○ At least once a month
- ○ At least once every 6 months
- ○ Only once in the year
- ○ No contact in the last year

You indicated you have had regular or seasonal contact with dogs in the **last 10 years**. What was the reason for this? *Select all that apply*

- ☐ Work
- ☐ Pet
- ☐ Hobby (e.g. smallholding)
- ☐ Other, please specify

In the last year, how often on average, have you been in contact with dogs?

- ○ Every day
- ○ At least once a week
- ○ At least once a month
- ○ At least once every 6 months
- ○ Only once in the year
- ○ No contact in the last year

You indicated you have had regular or seasonal contact with rabbits in the **last 10 years**. What was the reason for this? *Select all that apply*

- ☐ Work
- ☐ Pet
- ☐ Hobby (e.g. smallholding)
- ☐ Other, specify

In the last year, how often on average, have you been in contact with rabbits?

- ○ Every day
- ○ At least once a week
- ○ At least once a month
- ○ At least once every 6 months
- ○ Only once in the year
- ○ No contact in the last year

You indicated you have had regular or seasonal contact with guinea pigs in the **last 10 years**. What was the reason for this? *Select all that apply*

- ☐ Work
- ☐ Pet
- ☐ Hobby (e.g. smallholding)
- ☐ Other, please specify

In the last year, how often on average, have you been in contact with guinea pigs?

- ○ Every day
- ○ At least once a week
- ○ At least once a month
- ○ At least once every 6 months
- ○ Only once in the year
- ○ No contact in the last year

You indicated you have had regular or seasonal contact with rodents (e.g. mice, rats, hamsters, gerbils) in the **last 10 years**. What was the reason for this? *Select all that apply*

- ☐ Work
- ☐ Pet
- ☐ Hobby (e.g. smallholding)
- ☐ Other, please specify

[ ]

In the last year, how often on average, have you been in contact with rodents (e.g. mice, rats, hamsters, gerbils)?

- ○ Every day
- ○ At least once a week
- ○ At least once a month
- ○ At least once every 6 months
- ○ Only once in the year
- ○ No contact in the last year

You indicated you have had regular or seasonal contact with horses in the **last 10 years**. What was the reason for this? *Select all that apply*

- ☐ Work
- ☐ Pet
- ☐ Hobby (e.g. smallholding)
- ☐ Other, please specify

[ ]

In the last year, how often on average, have you been in contact with horses?

- ○ Every day
- ○ At least once a week
- ○ At least once a month
- ○ At least once every 6 months
- ○ Only once in the year
- ○ No contact in the last year

You indicated you have had regular or seasonal contact with pet birds in the **last 10 years**. What was the reason for this? *Select all that apply*

- ☐ Work
- ☐ Pet
- ☐ Hobby (e.g. smallholding)
- ☐ Other, please specify

In the last year, how often on average, have you been in contact with pet birds?

- ○ Every day
- ○ At least once a week
- ○ At least once a month
- ○ At least once every 6 months
- ○ Only once in the year
- ○ No contact in the last year

You indicated you have had regular or seasonal contact with reptiles in the **last 10 years**. What was the reason for this? *Select all that apply*

- ☐ Work
- ☐ Pet
- ☐ Hobby (e.g. smallholding)
- ☐ Other, please specify

In the last year, how often on average, have you been in contact with reptiles?

- ○ Every day
- ○ At least once a week
- ○ At least once a month
- ○ At least once every 6 months
- ○ Only once in the year
- ○ No contact in the last year

You indicated you have had regular or seasonal contact with cattle in the **last 10 years**. What was the reason for this? *Select all that apply*

- ☐ Work
- ☐ Pet
- ☐ Hobby (e.g. smallholding)
- ☐ Other, please specify

In the last year, how often on average, have you been in contact with cattle?

- ○ Every day
- ○ At least once a week
- ○ At least once a month
- ○ At least once every 6 months
- ○ Only once in the year
- ○ No contact in the last year

You indicated you have had regular or seasonal contact with pigs in the **last 10 years**. What was the reason for this? *Select all that apply*

- ☐ Work
- ☐ Pet
- ☐ Hobby (e.g. smallholding)
- ☐ Other, please specify

In the last year, how often on average, have you been in contact with pigs?

- ○ Every day
- ○ At least once a week
- ○ At least once a month
- ○ At least once every 6 months
- ○ Only once in the year
- ○ No contact in the last year

You indicated you have had regular or seasonal contact with sheep or goats in the **last 10 years**. What was the reason for this? *Select all that apply*

- ☐ Work
- ☐ Pet
- ☐ Hobby (e.g. smallholding)
- ☐ Other, please specify

In the last year, how often on average, have you been in contact with sheep or goats?

- ○ Every day
- ○ At least once a week
- ○ At least once a month
- ○ At least once every 6 months
- ○ Only once in the year
- ○ No contact in the last year

You indicated you have had regular or seasonal contact with poultry or reared gamebirds in the **last 10 years**. What was the reason for this? *Select all that apply*

- ☐ Work
- ☐ Pet
- ☐ Hobby (e.g. smallholding)
- ☐ Other, please specify

In the last year, how often on average, have you been in contact with poultry or reared gamebirds?

- ○ Every day
- ○ At least once a week
- ○ At least once a month
- ○ At least once every 6 months
- ○ Only once in the year
- ○ No contact in the last year

You indicated you have had regular or seasonal contact with deer in the **last 10 years**. What was the reason for this? *Select all that apply*

- ☐ Work
- ☐ Pet
- ☐ Hobby (e.g. smallholding)
- ☐ Other, specify

In the last year, how often on average, have you been in contact with deer?

- ○ Every day
- ○ At least once a week
- ○ At least once a month
- ○ At least once every 6 months
- ○ Only once in the year
- ○ No contact in the last year

You indicated you have had regular or seasonal contact with wild birds in the **last 10 years**. What was the reason for this? *Select all that apply*

- ☐ Work
- ☐ Pet
- ☐ Hobby (e.g. smallholding)
- ☐ Other, please specify

In the last year, how often on average, have you been in contact with wild birds?

- ○ Every day
- ○ At least once a week
- ○ At least once a month
- ○ At least once every 6 months
- ○ Only once in the year
- ○ No contact in the last year

You indicated you have had regular or seasonal contact with insects in the **last 10 years**. What was the reason for this? *Select all that apply*

- ☐ Work
- ☐ Pet
- ☐ Hobby (e.g. smallholding)
- ☐ Other, please specify

In the last year, how often on average, have you been in contact with insects?

- ○ Every day
- ○ At least once a week
- ○ At least once a month
- ○ At least once every 6 months
- ○ Only once in the year
- ○ No contact in the last year

You indicated you have had regular or seasonal contact with fish in the **last 10 years**. What was the reason for this? *Select all that apply*

- ☐ Work
- ☐ Pet
- ☐ Hobby (e.g. smallholding)
- ☐ Other, please specify

In the last year, how often on average, have you been in contact with fish?

- ○ Every day
- ○ At least once a week
- ○ At least once a month
- ○ At least once every 6 months
- ○ Only once in the year
- ○ No contact in the last year

You indicated you have had regular or seasonal contact with bats in the **last 10 years**. What was the reason for this? *Select all that apply*

- ☐ Work
- ☐ Pet
- ☐ Hobby (e.g. smallholding)
- ☐ Other, please specify

In the last year, how often on average, have you been in contact with bats?

- ○ Every day
- ○ At least once a week
- ○ At least once a month
- ○ At least once every 6 months
- ○ Only once in the year
- ○ No contact in the last year

You indicated you have had regular or seasonal contact with camels, llamas or alpacas in the **last 10 years**. What was the reason for this? *Select all that apply*

- ☐ Work
- ☐ Pet
- ☐ Hobby (e.g. smallholding)
- ☐ Other, please specify

In the last year, how often on average, have you been in contact with camels, llamas or alpacas?

- ○ Every day
- ○ At least once a week
- ○ At least once a month
- ○ At least once every 6 months
- ○ Only once in the year
- ○ No contact in the last year

You indicated you have had regular or seasonal contact with non-human primates (monkeys or apes) in the **last 10 years**. What was the reason for this? *Select all that apply*

- [ ] Work
- [ ] Pet
- [ ] Hobby (e.g. smallholding)
- [ ] Other, please specify

In the last year, how often on average, have you been in contact with non-human primates (monkeys or apes)?

- ○ Every day
- ○ At least once a week
- ○ At least once a month
- ○ At least once every 6 months
- ○ Only once in the year
- ○ No contact in the last year

You indicated you have had regular or seasonal contact with {Q88a} in the **last 10 years**. What was the reason for this? *Select all that apply*

- [ ] Work
- [ ] Pet
- [ ] Hobby (e.g. smallholding)
- [ ] Other, please specify

In the last year, how often on average, have you been in contact with {Q88a}?

- ○ Every day
- ○ At least once a week
- ○ At least once a month
- ○ At least once every 6 months
- ○ Only once in the year
- ○ No contact in the last year

You indicated you have had regular or seasonal contact with {Q88b} in the **last 10 years**. What was the reason for this? *Select all that apply*

- ☐ Work
- ☐ Pet
- ☐ Hobby (e.g. smallholding)
- ☐ Other, please specify

In the last year, how often on average, have you been in contact with {Q88b}?

- ○ Every day
- ○ At least once a week
- ○ At least once a month
- ○ At least once every 6 months
- ○ Only once in the year
- ○ No contact in the last year

You indicated you have had regular or seasonal contact with {Q88c} in the **last 10 years**. What was the reason for this? *Select all that apply*

- ☐ Work
- ☐ Pet
- ☐ Hobby (e.g. smallholding)
- ☐ Other, please specify

In the last year, how often on average, have you been in contact with {Q88c}?

- ○ Every day
- ○ At least once a week
- ○ At least once a month
- ○ At least once every 6 months
- ○ Only once in the year
- ○ No contact in the last year

You indicated you have had regular or seasonal contact with {Q88d} in the **last 10 years**. What was the reason for this? *Select all that apply*

- ☐ Work
- ☐ Pet
- ☐ Hobby (e.g. smallholding)
- ☐ Other, please specify

In the last year, how often on average, have you been in contact with {Q88d}?

- ○ Every day
- ○ At least once a week
- ○ At least once a month
- ○ At least once every 6 months
- ○ Only once in the year
- ○ No contact in the last year

You indicated you have had regular or seasonal contact with {Q88e} in the **last 10 years**. What was the reason for this? *Select all that apply*

- ☐ Work
- ☐ Pet
- ☐ Hobby (e.g. smallholding)
- ☐ Other, please specify

In the last year, how often on average, have you been in contact with {Q88e}?

- ○ Every day
- ○ At least once a week
- ○ At least once a month
- ○ At least once every 6 months
- ○ Only once in the year
- ○ No contact in the last year

You indicated you have had regular or seasonal contact with {Q88f} in the **last 10 years**. What was the reason for this? *Select all that apply*

- ☐ Work
- ☐ Pet
- ☐ Hobby (e.g. smallholding)
- ☐ Other, please specify

In the last year, how often on average, have you been in contact with {Q88f}?

- ◯ Every day
- ◯ At least once a week
- ◯ At least once a month
- ◯ At least once every 6 months
- ◯ Only once in the year
- ◯ No contact in the last year

You indicated you have had regular or seasonal contact with {Q88g} in the **last 10 years**. What was the reason for this? *Select all that apply*

- ☐ Work
- ☐ Pet
- ☐ Hobby (e.g. smallholding)
- ☐ Other, please specify

In the last year, how often on average, have you been in contact with {Q88g}?

- ◯ Every day
- ◯ At least once a week
- ◯ At least once a month
- ◯ At least once every 6 months
- ◯ Only once in the year
- ◯ No contact in the last year

You indicated you have had regular or seasonal contact with {Q88h} in the **last 10 years**. What was the reason for this? *Select all that apply*

- ☐ Work
- ☐ Pet
- ☐ Hobby (e.g. smallholding)
- ☐ Other, please specify

In the last year, how often on average, have you been in contact with {Q88h}?

- ○ Every day
- ○ At least once a week
- ○ At least once a month
- ○ At least once every 6 months
- ○ Only once in the year
- ○ No contact in the last year

You indicated you have had regular or seasonal contact with {Q88i} in the **last 10 years**. What was the reason for this? *Select all that apply*

- ☐ Work
- ☐ Pet
- ☐ Hobby (e.g. smallholding)
- ☐ Other, please specify

In the last year, how often on average, have you been in contact with {Q88i}?

- ○ Every day
- ○ At least once a week
- ○ At least once a month
- ○ At least once every 6 months
- ○ Only once in the year
- ○ No contact in the last year

You indicated you have had regular or seasonal contact with {Q88j} in the **last 10 years**. What was the reason for this? *Select all that apply*

- ☐ Work
- ☐ Pet
- ☐ Hobby (e.g. smallholding)
- ☐ Other, please specify

In the last year, how often on average, have you been in contact with {Q88j}?

- ◯ Every day
- ◯ At least once a week
- ◯ At least once a month
- ◯ At least once every 6 months
- ◯ Only once in the year
- ◯ No contact in the last year

In the last 10 years, have you been **bitten** by any of the following animals? *Select all that apply*

- ☐ No, I have not been bitten by an animal
- ☐ I cannot remember if I have been bitten by an animal
- ☐ Yes, by a dog
- ☐ Yes, by a non-human primate (monkeys or apes)
- ☐ Yes, by a cat
- ☐ Yes, by a bat
- ☐ Yes, by a reptile
- ☐ Yes by another animal

Please check your answer. You cannot select 'No, I have not been bitten by an animal' with another option

Please check your answer. You cannot select 'I cannot remember if I have been bitten by an animal' with another option

Specify what animal. Use a new box for each animal (4 boxes are available)

You indicated you were bitten by a dog. Was this in the UK and / or abroad? *Select all that apply*

- ☐ UK
- ☐ Abroad

Specify which country or countries abroad

When was your most recent dog bite?

- ◯ Within the last week
- ◯ Within the last month
- ◯ Within the last 6 months
- ◯ Within the last year
- ◯ More than 1 year ago

You indicated you were bitten by a non-human primate (monkeys or apes). Was this in the UK and / or abroad? *Select all that apply*

- ☐ UK
- ☐ Abroad

Specify which country or countries abroad

When was your most recent non-human primate (monkeys or apes) bite?

- ◯ Within the last week
- ◯ Within the last month
- ◯ Within the last 6 months
- ◯ Within the last year
- ◯ More than 1 year ago

You indicated you were bitten by a cat. Was this in the UK and / or abroad? *Select all that apply*

- ☐ UK
- ☐ Abroad

Specify which country or countries abroad

When was your most recent cat bite?

- Within the last week
- Within the last month
- Within the last 6 months
- Within the last year
- More than 1 year ago

You indicated you were bitten by a bat. Was this in the UK and / or abroad? *Select all that apply*

- UK
- Abroad

Specify which country or countries abroad

When was your most recent bat bite?

- Within the last week
- Within the last month
- Within the last 6 months
- Within the last year
- More than 1 year ago

You indicated you were bitten by a reptile. Was this in the UK and / or abroad? *Select all that apply*

- UK
- Abroad

Specify which country or countries abroad

When was your most recent reptile bite ?

- Within the last week
- Within the last month
- Within the last 6 months
- Within the last year
- More than 1 year ago

You indicated you were bitten by a {Q147a}. Was this in the UK and / or abroad? *Select all that apply*

- [ ] UK
- [ ] Abroad

Specify which country or countries abroad

When was your most recent {Q147a} bite?

- Within the last week
- Within the last month
- Within the last 6 months
- Within the last year
- More than 1 year ago

You indicated you were bitten by a {Q147b}. Was this in the UK and / or abroad? *Select all that apply*

- [ ] UK
- [ ] Abroad

Specify which country or countries abroad

When was your most recent {Q147b} bite?

- Within the last week
- Within the last month
- Within the last 6 months
- Within the last year
- More than 1 year ago

You indicated you were bitten by a {Q147c}. Was this in the UK and / or abroad? *Select all that apply*

- [ ] UK
- [ ] Abroad

Specify which country or countries abroad

When was your most recent {Q147c} bite?

- ◯ Within the last week
- ◯ Within the last month
- ◯ Within the last 6 months
- ◯ Within the last year
- ◯ More than 1 year ago

You indicated you were bitten by a {Q147d}. Was this in the UK and / or abroad? *Select all that apply*

- ☐ UK
- ☐ Abroad

Specify which country or countries abroad

When was your most recent {Q147d} bite?

- ◯ Within the last week
- ◯ Within the last month
- ◯ Within the last 6 months
- ◯ Within the last year
- ◯ More than 1 year ago

Food products

Do you have any dietary exclusions? *Select all that apply*

- ☐ No dietary exclusions
- ☐ No milk or dairy products
- ☐ No eggs or egg products
- ☐ No red meat or poultry
- ☐ No red meat, fish or poultry
- ☐ No animal products of any kind
- ☐ Other

Please check your answer. You cannot select 'No dietary exclusions' with another option

You answered 'Other'. Please specify what you exclude from your diet.

[ ]

Have you ever knowingly consumed unpasteurised milk or milk products (e.g. cheese) in the UK or abroad? Unpasteurised milk is raw milk that has not been treated. *Select all that apply*

- ☐ Yes, in the UK
- ☐ Yes, abroad
- ☐ No
- ☐ Not sure

Please check your answer. You cannot select 'No' with another option

Please check your answer. You cannot select 'Not sure' with another option

You answered 'Yes, abroad'. Please specify in which country(ies) you consumed unpasteurised milk or milk products

[ ]

Please specify which animal(s) the unpasteurised milk or milk products came from. *Select all that apply*

- ☐ Cow
- ☐ Goat
- ☐ Sheep
- ☐ Camel
- ☐ Buffalo
- ☐ I don't know

Please check your answer. You cannot select 'I don't know' with another option

Outdoor occupations

The following questions ask if your current job, or a job you have held in the last 10 years, require you to work outdoors regularly e.g. for 1 day a week throughout the year, or more frequently but only for part of the year e.g. seasonally.

Does your current job, or a job you have held in the **last 10 years**, require regular / seasonal outdoor work?

- ◯ Yes — Go to Q172
- ◯ No — Go to N39

Which of these outdoor job descriptions best describe your role, or a role you have held in the **last 10 years**? *Select all that apply*

- ☐ Wildlife ranger, deer management or professional deer stalker
- ☐ Forestry worker, forest ranger or park ranger
- ☐ Countryside management or conservation
- ☐ Gamekeeper, gamebird manager or professional beater
- ☐ Farmer, farm manager, farm worker or small holder
- ☐ Gardener or landscaper
- ☐ Outdoor pursuits instructor
- ☐ Environmental scientist or field ecologist
- ☐ Veterinarian
- ☐ Water worker, sewerage etc
- ☐ Fisherman
- ☐ Other outdoor occupation

Please specify occupation <u>using a new box for each occupation</u> (4 boxes are available)

You indicated you are, or have been a wildlife ranger, deer manager or professional deer stalker. How many years have you worked in this occupation?

- Less than 1 year
- 1 to 5 years
- 6 to 10 years
- More than 10 years

You indicated you are, or have been a forestry worker, forest ranger or park ranger. How many years have you worked in this occupation?

- Less than 1 year
- 1 to 5 years
- 6 to 10 years
- More than 10 years

You indicated you are, or have been employed in countryside management or conservation. How many years have you worked in this occupation?

- Less than 1 year
- 1 to 5 years
- 6 to 10 years
- More than 10 years

You indicated you are, or have been a gamekeeper, gamebird manager or professional beater. How many years have you worked in this occupation?

- Less than 1 year
- 1 to 5 years
- 6 to 10 years
- More than 10 years

You indicated you are, or have been a farmer, farm manager, farm worker or small holder. How many years have you worked in this occupation?

- Less than 1 year
- 1 to 5 years
- 6 to 10 years
- More than 10 years

You indicated you are, or have been a gardener or landscaper. How many years have you worked in this occupation?

- Less than 1 year
- 1 to 5 years
- 6 to 10 years
- More than 10 years

You indicated you are, or have been an outdoor pursuits instructor. How many years have you worked in this occupation?

- Less than 1 year
- 1 to 5 years
- 6 to 10 years
- More than 10 years

You indicated you are, or have been an environmental scientist or field ecologist. How many years have you worked in this occupation?

- Less than 1 year
- 1 to 5 years
- 6 to 10 years
- More than 10 years

You indicated you are, or have been a veterinarian. How many years have you worked in this occupation?

- Less than 1 year
- 1 to 5 years
- 6 to 10 years
- More than 10 years

You indicated you are, or have been a water or sewerage worker. How many years have you worked in this occupation?

- Less than 1 year
- 1 to 5 years
- 6 to 10 years
- More than 10 years

You indicated you are, or have been a fisherman. How many years have you worked in this occupation?

- Less than 1 year
- 1 to 5 years
- 6 to 10 years
- More than 10 years

You indicated you are, or have been employed as a {Q172a}. How many years have you worked in this occupation?

- Less than 1 year
- 1 to 5 years
- 6 to 10 years
- More than 10 years

You indicated you are, or have been employed as a {Q172b}. How many years have you worked in this occupation?

- Less than 1 year
- 1 to 5 years
- 6 to 10 years
- More than 10 years

You indicated you are, or have been employed as a {Q172c}. How many years have you worked in this occupation?

- Less than 1 year
- 1 to 5 years
- 6 to 10 years
- More than 10 years

You indicated you are, or have been employed as a {Q172d}. How many years have you worked in this occupation?

- Less than 1 year
- 1 to 5 years
- 6 to 10 years
- More than 10 years

Outdoor hobbies and leisure activities

The following questions ask if you regularly spend time outdoors for leisure or have done so in the last 10 years. This includes activities e.g. dog-walking that you may carry out throughout the year, or more seasonal activities e.g. camping that you've done for several years

In the **last 10 years**, have you regularly spent time outdoors for hobbies or recreational activities?

- ○ Yes — Go to Q189
- ○ No — Go to Q228

Over the **last 10 years**, which of the following hobbies or outdoor leisure activities have you taken part in? *Select all that apply*

- ☐ Urban walking, running or dog walking
- ☐ Rural walking, running or dog walking
- ☐ Orienteering
- ☐ Climbing or bouldering
- ☐ Off-road or mountain biking
- ☐ Cycling (on roads or marked tracks)
- ☐ Horse riding
- ☐ Golf
- ☐ Field sport e.g. cricket, football, rugby, etc
- ☐ Gardening / spending time in the garden
- ☐ Camping or attending festivals
- ☐ Forest or conservation volunteer
- ☐ Recreational shooting, hunting, deer stalking or beating
- ☐ Freshwater sports e.g. swimming, sailing, rowing, potholing, caving, fishing, canoeing, paddleboarding
- ☐ Sea sports e.g. swimming, sailing, rowing, fishing, canoeing, paddleboarding, surfing
- ☐ Other outdoor leisure activity

Please specify other outdoor leisure activities, using a new box for each activity (4 boxes are available)

[ ]

[ ]

How many years have you been urban walking, running or dog walking?

- Less than 1 year
- 1 to 5 years
- 6 to 10 years
- More than 10 years

In the last year, how often on average, do you go urban walking, running or dog walking?

- Every day
- At least once a week
- At least once a month
- At least once every 6 months
- Only once in the year
- Not at all

How many years have you been rural walking, running or dog walking?

- Less than 1 year
- 1 to 5 years
- 6 to 10 years
- More than 10 years

In the last year, how often on average, do you go rural walking, running or dog walking?

- Every day
- At least once a week
- At least once a month
- At least once every 6 months
- Only once in the year
- Not at all

How many years have you been orienteering?

- Less than 1 year
- 1 to 5 years
- 6 to 10 years
- More than 10 years

In the last year, how often on average, do you go orienteering?

- Every day
- At least once a week
- At least once a month
- At least once every 6 months
- Only once in the year
- Not at all

How many years have you been climbing or bouldering?

- Less than 1 year
- 1 to 5 years
- 6 to 10 years
- More than 10 years

In the last year, how often on average, do you go climbing or bouldering?

- Every day
- At least once a week
- At least once a month
- At least once every 6 months
- Only once in the year
- Not at all

How many years have you been off-road or mountain biking?

- Less than 1 year
- 1 to 5 years
- 6 to 10 years
- More than 10 years

In the last year, how often on average, do you go off-road or mountain biking?

- Every day
- At least once a week
- At least once a month
- At least once every 6 months
- Only once in the year
- Not at all

How many years have you been cycling (on roads or marked tracks)?

- Less than 1 year
- 1 to 5 years
- 6 to 10 years
- More than 10 years

In the last year, how often on average, do you go cycling (on roads or marked tracks)?

- Every day
- At least once a week
- At least once a month
- At least once every 6 months
- Only once in the year
- Not at all

How many years have you been horse riding?

- Less than 1 year
- 1 to 5 years
- 6 to 10 years
- More than 10 years

In the last year, how often on average, do you go horse riding?

- Every day
- At least once a week
- At least once a month
- At least once every 6 months
- Only once in the year
- Not at all

How many years have you been playing golf?

- Less than 1 year
- 1 to 5 years
- 6 to 10 years
- More than 10 years

In the last year, how often on average, do you play golf?

- ◯ Every day
- ◯ At least once a week
- ◯ At least once a month
- ◯ At least once every 6 months
- ◯ Only once in the year
- ◯ Not at all

How many years have you been playing a field sport (e.g. cricket, football, rugby)?

- ◯ Less than 1 year
- ◯ 1 to 5 years
- ◯ 6 to 10 years
- ◯ More than 10 years

In the last year, how often on average, do you play a field sport e.g. cricket, football or rugby?

- ◯ Every day
- ◯ At least once a week
- ◯ At least once a month
- ◯ At least once every 6 months
- ◯ Only once in the year
- ◯ Not at all

How many years have you been gardening or spending time in the garden?

- ◯ Less than 1 year
- ◯ 1 to 5 years
- ◯ 6 to 10 years
- ◯ More than 10 years

In the last year, how often on average, do you garden or spend time in the garden?

- ◯ Every day
- ◯ At least once a week
- ◯ At least once a month
- ◯ At least once every 6 months
- ◯ Only once in the year
- ◯ Not at all

How many years have you been camping or attending festivals?

- ◯ Less than 1 year
- ◯ 1 to 5 years
- ◯ 6 to 10 years
- ◯ More than 10 years

In the last year, how often on average, do you go camping or attend festivals?

- ◯ Every day
- ◯ At least once a week
- ◯ At least once a month
- ◯ At least once every 6 months
- ◯ Only once in the year
- ◯ Not at all

How many years have you been a forest or conservation volunteer?

- ◯ Less than 1 year
- ◯ 1 to 5 years
- ◯ 6 to 10 years
- ◯ More than 10 years

In the last year, how often on average, do you do voluntary conservation or forestry work?

- ◯ Every day
- ◯ At least once a week
- ◯ At least once a month
- ◯ At least once every 6 months
- ◯ Only once in the year
- ◯ Not at all

How many years have you been a recreational shooter, hunter, deer stalker or beater?

- ◯ Less than 1 year
- ◯ 1 to 5 years
- ◯ 6 to 10 years
- ◯ More than 10 years

In the last year, how often on average, do you shoot, hunt, deer stalk or beat recreationally?

- ◯ Every day
- ◯ At least once a week
- ◯ At least once a month
- ◯ At least once every 6 months
- ◯ Only once in the year
- ◯ Not at all

How many years have you taken part in freshwater sports?

- ◯ Less than 1 year
- ◯ 1 to 5 years
- ◯ 6 to 10 years
- ◯ More than 10 years

In the last year, how often on average, do you take part in freshwater sports?

- ◯ Every day
- ◯ At least once a week
- ◯ At least once a month
- ◯ At least once every 6 months
- ◯ Only once in the year
- ◯ Not at all

How many years have you taken part in sea sports?

- ◯ Less than 1 year
- ◯ 1 to 5 years
- ◯ 6 to 10 years
- ◯ More than 10 years

In the last year, how often on average, do you take part in sea sports?

- ◯ Every day
- ◯ At least once a week
- ◯ At least once a month
- ◯ At least once every 6 months
- ◯ Only once in the year
- ◯ Not at all

You specified another outdoor hobby or activity - {Q189a}. How many years have you been doing this?

- Less than 1 year
- 1 to 5 years
- 6 to 10 years
- More than 10 years

In the last year, how often on average, do you take part in {Q189a}?

- Every day
- At least once a week
- At least once a month
- At least once every 6 months
- Only once in the year
- Not at all

You specified another outdoor hobby or activity - {Q189b}. How many years have you been doing this?

- Less than 1 year
- 1 to 5 years
- 6 to 10 years
- More than 10 years

In the last year, how often on average, do you take part in {Q189b}?

- Every day
- At least once a week
- At least once a month
- At least once every 6 months
- Only once in the year
- Not at all

You specified another outdoor hobby or activity - {Q189c}. How many years have you been doing this?

- Less than 1 year
- 1 to 5 years
- 6 to 10 years
- More than 10 years

In the last year, how often on average, do you take part in {Q189c}?

- Every day
- At least once a week
- At least once a month
- At least once every 6 months
- Only once in the year
- Not at all

You specified another outdoor hobby or activity - {Q189d}. How many years have you been doing this?

- Less than 1 year
- 1 to 5 years
- 6 to 10 years
- More than 10 years

In the last year, how often on average, do you take part in {Q189d}?

- Every day
- At least once a week
- At least once a month
- At least once every 6 months
- Only once in the year
- Not at all

If you would like to give more detail about your answers to any of the questions in this survey or have any other comments, please type them in the box below

You have completed the questionnaire! **Be sure to press the Submit button NOW**

Thank you for your time. The information you have provided will help us monitor the emergence of new and existing infectious diseases in England.

If you have any problems with this questionnaire, please contact CODONET@ukhsa.gov.uk
